# National Cardiovascular Data Registry-Acute Kidney Injury (NCDR) vs. Mehran risk models for prediction of contrast-induced nephropathy and need for dialysis after coronary angiography in a German patient cohort

**DOI:** 10.1007/s40620-021-01124-9

**Published:** 2021-08-07

**Authors:** Claudio Parco, Maximilian Brockmeyer, Lucin Kosejian, Julia Quade, Jennifer Tröstler, Selina Bader, Yingfeng Lin, Alexander Sokolowski, Alexander Hoss, Yvonne Heinen, Volker Schulze, Andrea Icks, Christian Jung, Malte Kelm, Georg Wolff

**Affiliations:** 1 Division of Cardiology, Pulmonology and Vascular Medicine, Department of Internal Medicine, Medical Faculty and University Hospital, Heinrich-Heine-University, Moorenstr. 5, 40225 Düsseldorf, Germany; 2 Institute for Health Services Research and Health Economics, Medical Faculty and University Hospital, Heinrich-Heine-University, Moorenstr. 5, 40225 Düsseldorf, Germany

**Keywords:** NCDR, Mehran, Risk prediction, Acute kidney injury

## Abstract

**Background:**

Contrast-induced nephropathy (CIN) is a major adverse event in patients undergoing coronary angiography. The Mehran risk model is the gold-standard for CIN risk prediction. However, its performance in comparison to more contemporary National Cardiovascular Data Registry-Acute Kidney Injury (NCDR-AKI) risk models remains unknown. We aimed to compare both in this study.

**Methods and results:**

Predictions of Mehran and NCDR-AKI risk models and clinical events of CIN and need for dialysis were assessed in a total of 2067 patients undergoing coronary angiography with or without percutaneous coronary intervention. Risk models were compared regarding discrimination (receiver operating characteristic analysis), net reclassification improvement (NRI) and calibration (graphical and statistical analysis). The NCDR risk model showed superior risk discrimination for predicting CIN (NCDR c-index 0.75, 95% CI 0.72–0.78; vs. Mehran c-index 0.69, 95% CI 0.66–0.72, p < 0.01), and continuous NRI (0.22; 95% CI 0.12–0.32; p < 0.01) compared to the Mehran model. The NCDR risk model tended to underestimate the risk of CIN, while the Mehran model was more evenly calibrated. For the prediction of need for dialysis, NCDR-AKI-D also discriminated risk better (c-index 0.85, 95% CI 0.79–0.91; vs. Mehran c-index 0.75, 95% CI 0.66–0.84; p_NCDRvsMehran_ < 0.01), but continuous NRI showed no benefit and calibration analysis revealed an underestimation of dialysis risk.

**Conclusion:**

In German patients undergoing coronary angiography, the modern NCDR risk model for predicting contrast-induced nephropathy showed superior discrimination compared to the Mehran model while showing less accurate calibration. Results for the outcome ‘need for dialysis’ were equivocal.

**Graphic abstract:**

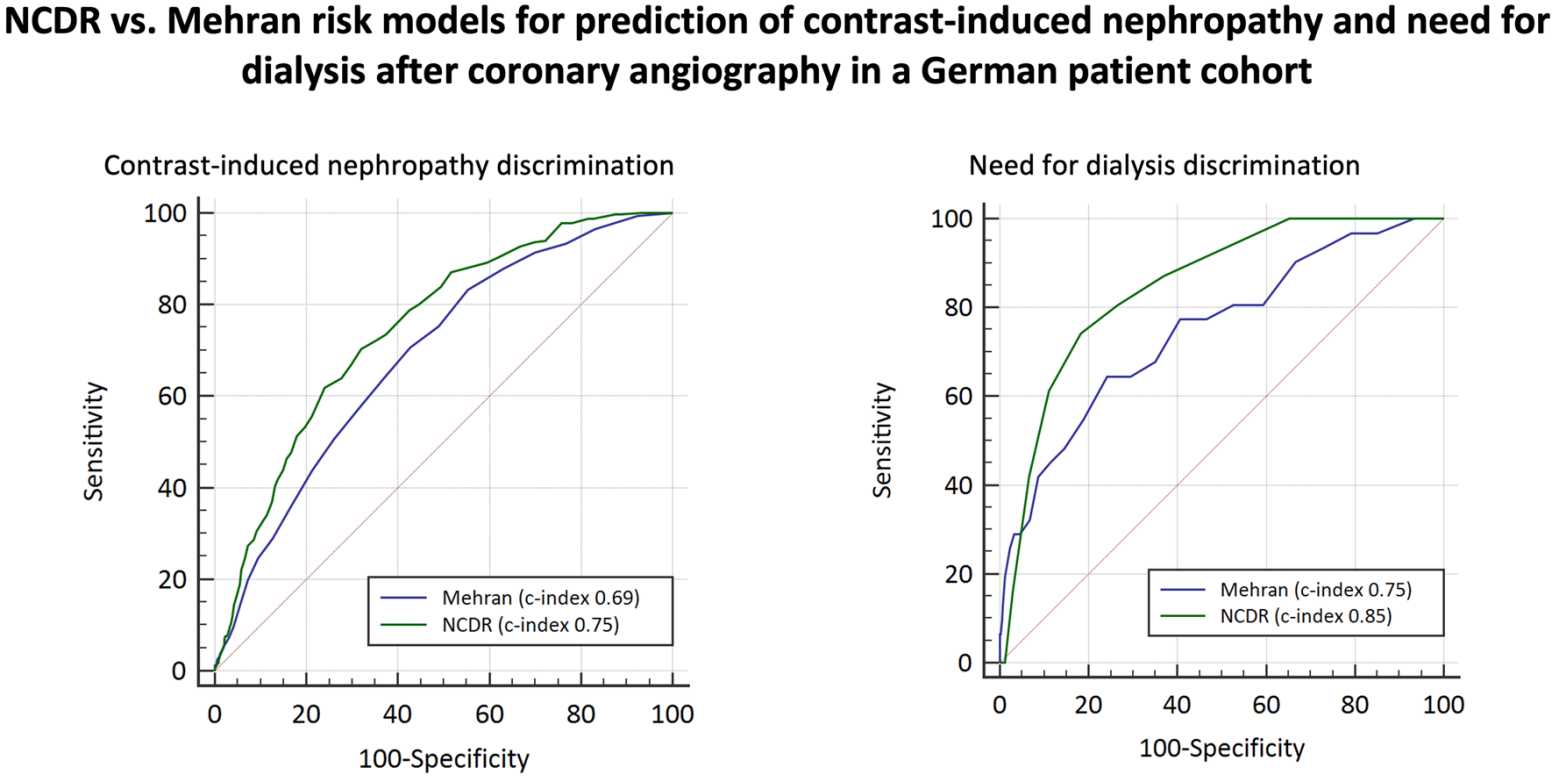

**Supplementary Information:**

The online version contains supplementary material available at 10.1007/s40620-021-01124-9.

## Introduction

Contrast-induced nephropathy (CIN) is a major adverse event for patients undergoing cardiac catheterization and is associated with increased mortality [1, 2]. CIN is defined as an impairment of renal function measured by an absolute (> 0.5 mg/dl) or relative (> 25%) increase in serum creatinine within 48–72 h after application of iodinated contrast media [3]. CIN after coronary angiography heralds a higher patient risk for long-term decline in renal function [4] and thus has profound prognostic relevance.

Several risk factors have been found associated with an increased risk of developing CIN [5]. Taking the most important risk factors (shock, congestive heart failure, older age, anemia, diabetes, contrast media volume, use of an intra-aortic balloon pump and chronic kidney disease) into account, in 2004 Mehran et al. developed a simple risk score to estimate the risk of developing CIN and need for dialysis [6]. To date, the original score has been validated in several cohorts [7, 8] and is the most established risk model for predicting CIN and need for dialysis. However, the more contemporary risk models of the National Cardiovascular Data Registry-Acute kidney Injury (NCDR-AKI) and NCDR-AKI-Dialysis (NCDR-AKI-D) based on data derived from 900,000 patients undergoing percutaneous coronary interventions (PCI) in the NCDR CathPCI Registry [9] showed promising performance in our external validation study [10]. The NCDR-AKI and NCDR-AKI-D models stratify the patients’ risk of developing contrast-CIN by the following variables; age, acute decompensated heart failure, glomerular filtration rate, and diabetes, history of cardiovascular disease and heart failure, cardiogenic shock, cardiac arrest, anemia, use of an intra-aortic balloon pump.

Overall, identifying patients at risk and adequate preventive management remain key factors for improving patient outcomes. We thus aimed to compare the modern NCDR risk models to the established Mehran model for predicting contrast-induced nephropathy in a contemporary German patient cohort undergoing cardiac catheterization procedures with and without percutaneous coronary intervention.

## Methods

### Patient characteristics, clinical settings and data collection

Patients undergoing invasive coronary angiography at University Hospital Duesseldorf between 2014 and 2018 for reasons ranging from elective cardiac catheterization procedures to presentation with acute non-ST-segment elevation myocardial infarction (NSTEMI) and ST-segment elevation myocardial infarction (STEMI) were included in the study. All patients were treated according to current European guideline-recommended clinical practice for acute and chronic coronary syndromes [11–14]. Patient and procedural characteristics, as well as symptoms and results diagnostic testing were extracted from medical records and recorded into a dedicated database. History of chronic kidney disease was defined as a previous decline in renal function according to the current Kidney Disease: Improving Global Outcomes (KDIGO) chronic kidney disease guidelines [15].

### Clinical outcomes definitions

The main outcomes of interest were CIN and need for dialysis. CIN was defined as an absolute increase in creatinine of more than 0.5 mg/dl or more than 25% compared to the baseline value within 48–72 h after cardiac catheterization [6]. Need for dialysis was defined as renal replacement therapy due to insufficient urine output, high retention parameters, metabolic acidosis or relevant electrolyte disorders during hospitalization. CIN events in patients who underwent chronic renal replacement therapy were not considered. Secondary outcomes, e.g. in-hospital mortality, major bleeding or stroke are additionally reported (Table 2).

### Statistics

Data collection and descriptive statistics were done using Access (Microsoft), Excel (Microsoft) and SPSS 26 (IBM). Ordinal/categorical variables are presented as counts and % of total, continuous data are presented as means ± standard deviation (SD). Statistical significance was assumed at a two-sided α probability < 0.05 for all analyses.

#### Missing data

Missing data were imputed only if they were relevant for risk model calculation, either to the most common value (binary/ordinal/categorical variables) or to the mean (continuous variables) of the respective clinical setting subgroup (NSTEMI, STEMI or elective). Details on missing variables as % of all model-relevant variables are reported in the Results section. Patients lacking information on relevant outcome variables (CIN or need for dialysis) were excluded. Sensitivity analysis of patients with complete datasets was additionally performed (no imputation).

#### Risk model calculation

The Mehran B risk model was calculated to predict CIN and the need for dialysis [6]. The NCDR-AKI risk model was calculated to predict CIN [9], while the NCDR-AKI-D model was used to predict the need for dialysis [9]. For the calculation of individual risk scores, all relevant scoring parameters (Suppl. Tables 1–3) were weighted according to risk model definitions and summarized to obtain an individual summary risk score for each patient. Summary risk scores were assigned to event probabilities according to risk model definitions ([6, 9], Suppl. Tables 1–3).

#### Risk model discrimination analysis

Risk model discrimination performance was analyzed using receiver operating characteristics (ROC) curves with area under the curve (AUC, c-index) comparisons using the non-parametric DeLong method [16]. All calculations were done using MedCalc v18.21 (MedCalc Software, Belgium). C-indices with 95% confidence intervals are reported [17, 18].

#### Risk model reclassification analysis

Reclassification of patients with the NCDR risk models compared to the standard Mehran model was evaluated using the package PredictABEL for R Studio v4.0.3: net reclassification improvements (NRI; categorical and continuous) were calculated [19, 20] and tested for statistical significance. For categorical NRI, all patients were classified into arbitrary categories: predicted CIN risk of 0–10% (low risk), 10–20% (intermediate risk) and 20–100% (high-risk) and predicted dialysis risk of 0–1% (low risk, 1–5% (intermediate risk) and 5–100% (high risk) (Suppl. Tab. 4 and 5); both were tested for significant reclassification between groups.

#### Risk model calibration analysis

Risk model calibration/goodness-of-fit was graphically analyzed [GraphPad Prism 8 (GraphPad Software Inc.)] comparing observed events for patient quintiles of predicted risk based on historical event probabilities from the original derivation cohorts [6, 9]; it was formally tested with a logistic regression model and—where possible—the Hosmer–Lemeshow goodness-of-fit test [21] (MedCalc v18.21 (MedCalc Software, Belgium). For the Mehran risk model for predicting contrast-induced nephropathy, assignment of score values to predicted risk were done according to extracted data from the central figure of the development cohort in the original publication [6].

## Results

### Patient and procedural characteristics

A total of 2,067 patients undergoing coronary angiography between 2014 and 2018 with and without PCI at University Hospital Duesseldorf were included. Patient characteristics and NCDR risk model performance indices of a sub-cohort of these patients (n = 1637) have been previously published by our group [10]. Patients were divided into clinical setting subgroups of elective, NSTEMI and STEMI.

Mean age in the overall cohort was 69 ± 12.3 years, 27.1% of the patients had a history of chronic kidney disease, 2.9% were treated with chronic dialysis. PCI was performed in 59.5% of all patients, the rest underwent diagnostic procedures alone. Mean applied contrast volume was 154.7 ± 97.0 ml, most in STEMI and least in elective procedures. Mechanical circulatory support was used in 2.3% (Impella®) and 1.9% (Extracorporeal life support, ECLS), respectively. The predominant symptom at admission was angina pectoris. Patient and procedural characteristics for all patients and for respective subgroups are reported in Table 1.

**Table 1:** Patient characteristics, comorbidities, symptoms at admission and procedural characteristics (left column) and separately for patient subsets of NSTEMI, STEMI and elective procedures

	All (n = 2067)	NSTEMI (n = 1002, 48%)	STEMI (n = 565, 27%)	Elective (n = 500, 24%)
Patient characteristics				
Age (y)	69.2 ± 12.3	72.2 ± 11.3	65.1 ± 13.4	67.7 ± 11.3
Body Mass Index (BMI)	27.5 ± 5.1	27.6 ± 5.3	26.7 ± 4.8	28.1 ± 4.8
Male sex	1439 (69.6)	700 (69.9)	399 (70.7)	340 (68.0)
Diabetes mellitus	608 (29.4)	365 (36.4)	95 (16.8)	148 (29.6)
Chronic kidney disease (eGFR < 60 ml/min)	560 (27.1)	323 (32.2)	158 (28.0)	79 (15.8)
Chronic dialysis	60 (2.9)	43 (4.3)	11 (1.9)	6 (1.2)
Coronary artery disease	948 (45.9)	507 (50.6)	107 (19.0)	334 (66.8)
Prior coronary artery bypass grafting (CABG)	191 (9.2)	153 (15.3)	4 (0.7)	34 (6.8)
Prior percutaneous coronary intervention (PCI)	611 (29.6)	309 (30.8)	20 (3.5)	282 (56.4)
History of heart failure	638 (30.9)	473 (47.2)	32 (5.7)	133 (26.6)
Anemia	622 (30.1)	375 (37.4)	156 (27.6)	91 (18.2)
Symptoms at admission				
Unstable angina	674 (32.6)	391 (39.0)	263 (46.5)	20 (4.0)
Dyspnea NYHA IV	191 (9.2)	124 (12.4)	56 (9.9)	11 (2.2)
Cardiac arrest within 24 h	76 (3.7)	32 (3.2)	44 (7.8)	0
Endotracheal intubation	58 (2.8)	37 (3.7)	21 (3.7)	0
Cardiogenic shock	144 (7.0)	72 (7.2)	72 (12.7)	0
Procedural characteristics				
PCI performed	1230 (59.5)	519 (51.8)	533 (94.3)	178 (35.6)
Contrast media volume (ml)	154.7 ± 97.0	148.9 ± 86.7	209.9 ± 105.8	107.3 ± 75.7
Procedure duration (min)	54.9 ± 33.9	56.1 ± 29.6	65.5 ± 43.4	41.0 ± 24.3
Impella® mechanical support	47 (2.3)	20 (2.0)	27 (4.8)	1 (0.2)
Extracorporeal life support	40 (1.9)	4 (0.4)	36 (6.4)	0

Data are presented as n (%) or as mean ± SD, unless specified differently

*eGFR* estimated glomerular filtration rate, *(N)STEMI* (Non) ST-segment elevation myocardial infarction, *NYHA* New York Heart Association

### Contrast-induced nephropathy outcomes

Contrast-induced nephropathy occurred in 15.3% (n = 317) of all patients, 1.5% (n = 31) consecutively required renal replacement therapy. While CIN was relatively common in patients presenting with acute coronary syndrome (21.3% in NSTEMI patients, 17.9% in STEMI patients), only 0.6% of all patients undergoing elective procedures developed CIN, none of whom required dialysis. In-hospital clinical outcomes are reported in Table 2.

**Table 2:** Overview of in-hospital clinical outcomes

	All (n = 2067)	NSTEMI (n = 1002, 48%)	STEMI (n = 565, 27%)	Elective (n = 500, 24%)
Contrast-induced nephropathy	317 (15.3)	213 (21.3)	101 (17.9)	3 (0.6)
Need for dialysis	31 (1.5)	15 (1.5)	16 (2.8)	0
All-cause mortality	119 (5.8)	37 (3.7)	82 (14.5)	0
Cardiovascular mortality	81 (3.9)	28 (2.8)	53 (9.4)	0
Major bleeding	120 (5.8)	59 (5.9)	60 (10.6)	1 (0.2)
Stroke	6 (0.3)	3 (0.3)	3 (0.5)	0

Data are presented as n (%)

*(N)STEMI* (Non) ST-segment elevation myocardial infarction

### Risk model performance evaluation

#### Missing data

For the calculation of the Mehran risk model, 3.8% missing values were imputed for the following variables; hematocrit (n = 11), contrast media volume (n = 108), glomerular filtration rate (n = 15), congestive heart failure (n = 1), systolic blood pressure (n = 493). For the calculation of the NCDR risk model, 0.12% missing values were imputed for these variables; hemoglobin (n = 11), glomerular filtration rate (n = 15), prior heart failure (n = 1).

#### Risk model discrimination (Fig. 1 and Table 3)

**Fig. 1:**
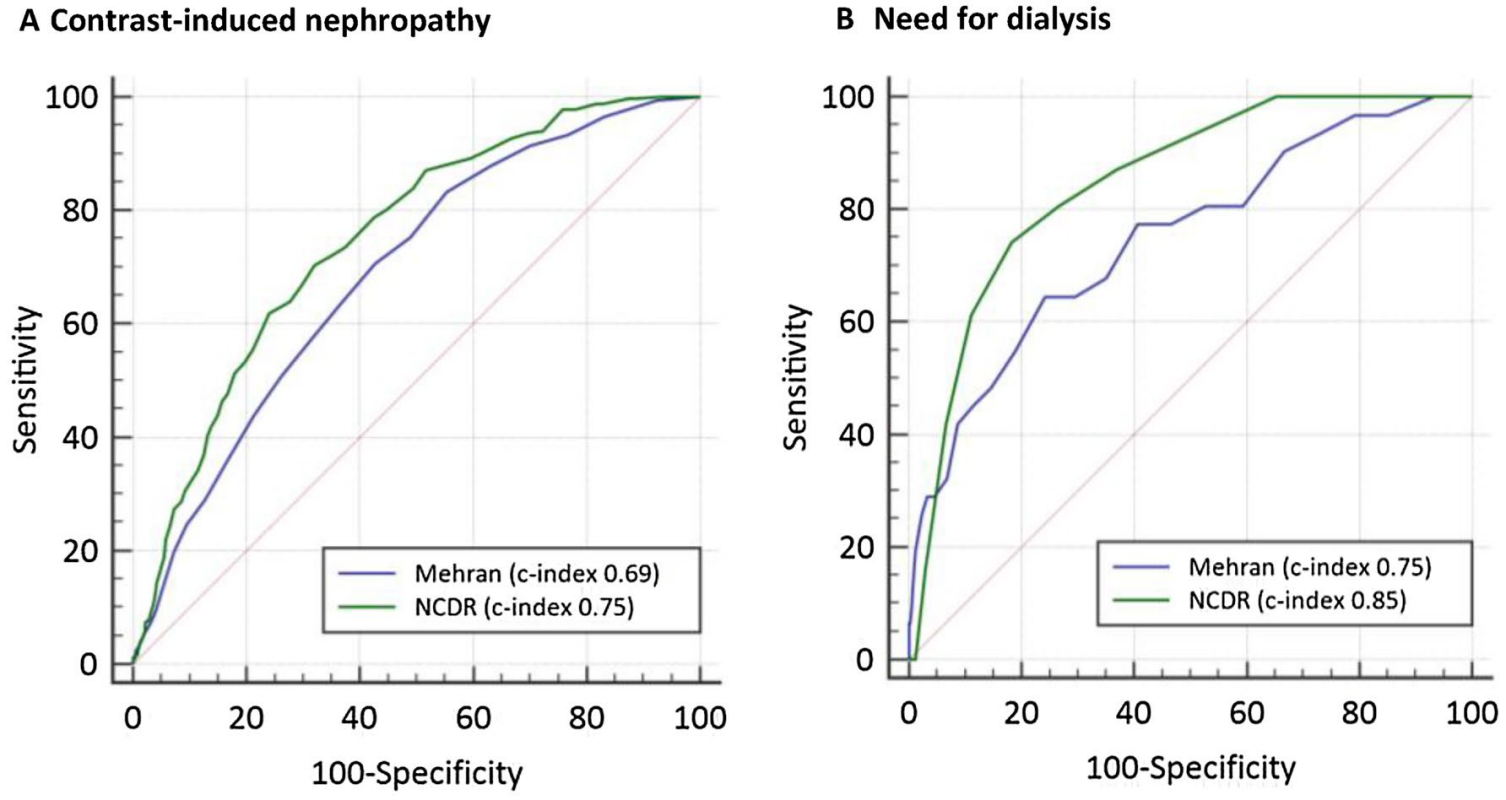
Comparative risk model discrimination performance analysis of ROC curves of National Cardiovascular Data Registry (NCDR) and Mehran risk models for A contrast induced nephropathy and B need for dialysis. Statistical comparisons were performed using the DeLong method [16], results are also reported in Table 3

**Table 3:** Comparative risk model performance analysis regarding model discrimination (a), reclassification (b) and calibration (c) for contrast-induced nephropathy and need for dialysis

	Contrast-induced nephropathy	Need for dialysis
Observed outcome events	317 (15.3%)	31 (1.5%)
Risk model discrimination: areas-under-curve/c-indices		
NCDR-AKI/NCDR AKI-D	0.75 (0.72–0.78)	0.85 (0.79–0.91)
Mehran	0.69 (0.66–0.72)	0.75 (0.66–0.84)
Statistics	p < 0.01	p < 0.01
Risk model reclassification: continuous net reclassification improvement		
Mehran vs. NCDR-AKI/NCDR AKI-D	0.22 (0.12–0.32)	− 0.16 (− 0.50–0.18)
	p < 0.01	p = 0.35
Risk model calibration: cohort mean risk prediction		
NCDR-AKI/NCDR AKI-D	11.6 ± 10.6%	0.3 ± 0.7%
Mehran	17.7 ± 14.0%	1.4 ± 3.6%

Model discrimination is reported as areas-under-curve (AUC, c-indices) of receiver-operating characteristic (ROC) analyses with 95% confidence intervals; reclassification is reported as continuous net reclassification improvement (NRI) with 95% confidence intervals; mean risk prediction is reported as mean ± standard deviation; p < 0.05 was considered statistically significant

*NCDR* National Cardiovascular Data Registry

The NCDR risk models showed good discrimination of risk for CIN (c-index 0.75, 95% CI 0.72–0.78) and very accurate performance in predicting need for dialysis (c-index 0.85, 95% CI 0.79–0.91). The Mehran risk model showed mediocre performance for CIN (c-index 0.69, 95% CI 0.66–0.72) and good discrimination for need for dialysis (c-index 0.75, 0.66–0.84). For both outcomes, the NCDR-AKI and NCDR-AKI-D risk models performed superior to the Mehran model (p_NCDRvsMehran_ < 0.01). The sensitivity analysis including only patients with complete datasets (no imputation, n = 1483) confirmed these results for CIN (c-index 0.70; 95% CI 0.67–0.74 (NCDR) vs. c-index 0.66; 95% CI 0.62–0.69 (Mehran); p < 0.01) and for need for dialysis (c-index 0.81; 95% CI 0.73–0.89 (NCDR) vs. 0.71; 95% CI 0.59–0.83 (Mehran); p = 0.03).

#### Risk model reclassification (Table 3 and Suppl. Tab. 4 + 5)

Reclassification analyses revealed a significant continuous NRI for the NCDR risk model compared to the Mehran risk model of 0.22 (95% CI 0.12–0.32; p < 0.01; Table 3) for CIN, which was confirmed in patients with complete datasets alone (sensitivity analysis: continuous NRI 0.19; 95% CI 0.07–0.31; p < 0.01). The continuous NRI for need for dialysis was not significant (NRI: − 0.16; 95% CI − 0.50 to 0.18; p = 0.35). Categorical NRI analysis on CIN did not reveal significant differences between models (Suppl. Tab. 4), while Mehran classified better than NCDR-AKI-D for the chosen risk categories in need for dialysis (Suppl. Tab. 5).

#### Risk model calibration (Fig. 2 and Table 3)

**Fig. 2:**
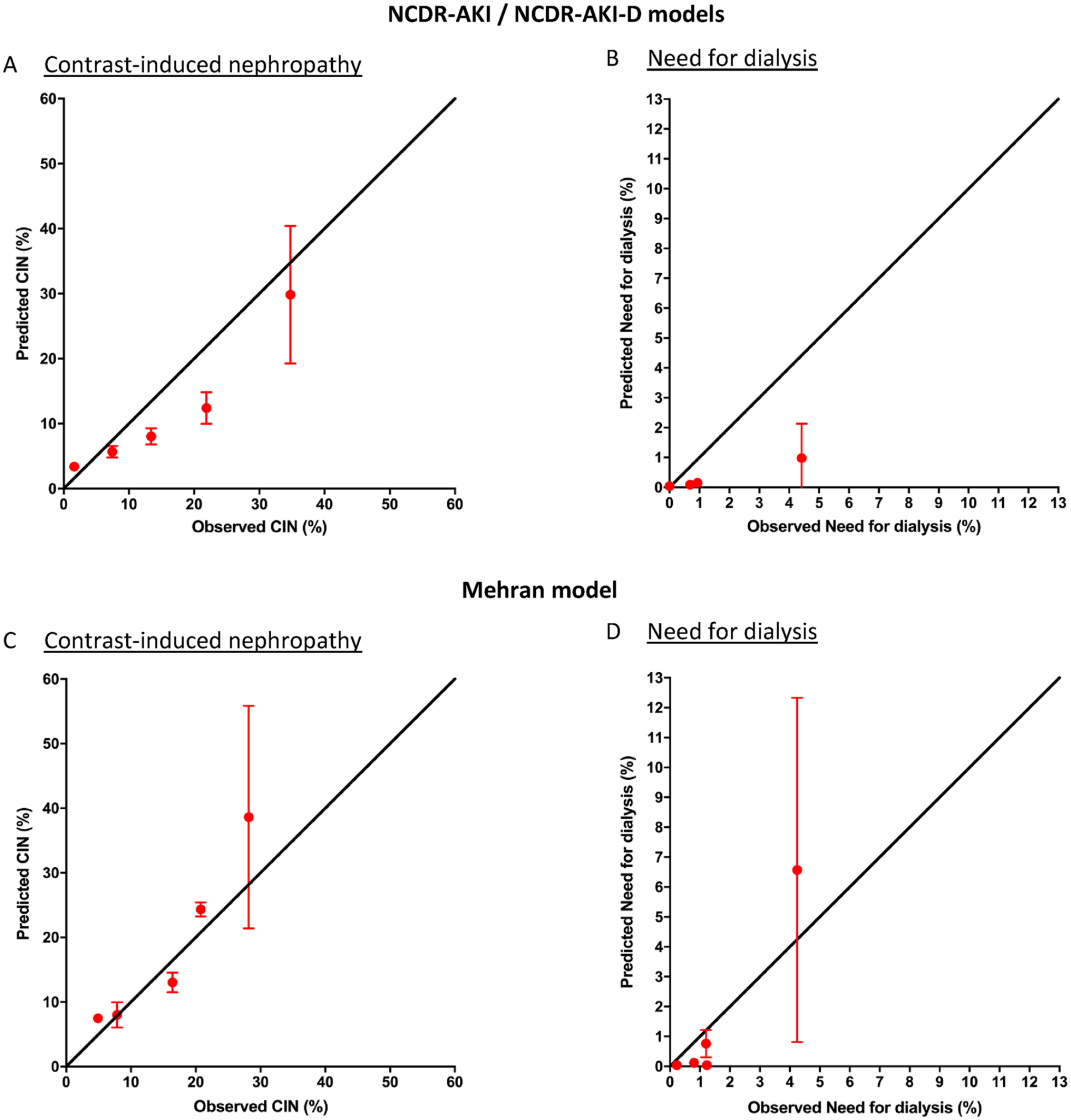
Risk model calibration for the National Cardiovascular Data Registry-Acute Kidney Injury (NCDR-AKI) and Dialysis (NCDR-AKI-D) and Mehran risk models, comparing observed and predicted contrast-induced nephropathy and need for dialysis in risk quintiles of all patients. *CIN* contrast-induced nephropathy

In the graphical analysis (Fig. 2A and C), the NCDR risk model tended to underestimate risk, especially in intermediate risk quintiles, while showing good calibration in high-risk patients. The Mehran risk model showed more accurate calibration in the graphical calibration analysis and only overestimated risk in the highest risk quintile. The Hosmer–Lemeshow goodness-of-fit test showed p < 0.01 for the NCDR-AKI as well as the Mehran risk model for predicting contrast-induced nephropathy. Both risk models showed inaccurate calibration for the outcome of need for dialysis (Fig. 2B and D). The Hosmer–Lemeshow goodness-of-fit test showed p < 0.01 for the NCDR-AKI-D model.

## Discussion

We herein present a comparative performance evaluation of NCDR and Mehran risk models for predicting contrast-induced nephropathy and need for dialysis in patients undergoing coronary angiography for elective and emergency indications. Main results are: (1) NCDR risk models for predicting CIN and need for dialysis (NCDR-AKI and NCDR-AKI-D) discriminated risk superior to the Mehran model; (2) NCDR CIN prediction showed significant continuous reclassification improvement compared to the Mehran model; (3) Both risk models lacked calibration, especially in dialysis risk prediction. However, the Mehran risk model for predicting contrast-induced nephropathy showed slightly better calibration in the graphical analysis while the NCDR risk model tended to underestimate risk for CIN.

Over the past decades, interventional cardiology has undergone tremendous changes in technology [22], pharmacological therapy [23–25] and demographic characteristics of patients [26]. But indications for invasive coronary angiography are also changing: interventional management of chronic coronary syndromes has become an increasingly controversial topic, taking the latest results from the ISCHEMIA trial and a large meta-analysis into account [27, 28]. Thus, adequate risk assessment and management is an important part of daily clinical practice to improve patient outcomes. Temporal and external validation of established risk scores is necessary to optimize risk management and retain risk model performance. Standard risk scores developed in patient cohorts almost two decades ago – like the Mehran risk model – might not be the most accurate option anymore.

The Mehran risk model for predicting contrast-induced nephropathy is well-established and was developed in 2004 by Mehran et al. in a cohort of 8357 patients [6]. The initial validation cohort showed similar risk discrimination performance (c-index of 0.67 [6]) for contrast-induced nephropathy compared to our cohort (c-index of 0.69). For predicting need for dialysis, the Mehran risk model also showed a reasonable discrimination performance (c-index 0.75). Graphical analysis revealed slightly better calibration for predicting contrast-induced nephropathy compared to the NCDR-AKI risk model, potentially due to similar rates of CIN (15.3% in our cohort vs. 13.1% in the development cohort [6]). Reasons for the inaccurate calibration regarding the outcome ‘need for dialysis’ may potentially lie in a limited number of events in our cohort (n = 31), a higher number of patients presenting with acute myocardial infarction (75.8% vs. 35.7% in the development cohort) and an overall older patient cohort (69.2 years vs. 63.8 years in the development cohort).

The National Cardiovascular Data Registry risk models for predicting acute kidney injury and need for dialysis were developed in a patient cohort of over 900,000 patients [9]. NCDR-AKI and NCDR-AKI-D showed good discrimination performance in the initial validation cohort with c-indices of 0.71 for NCDR-AKI and of 0.89 for NCDR-AKI-D [9]. We herein observed comparable results for predicting CIN (c-index 0.75) and need for dialysis (c-index 0.85). Calibration showed an underestimation of risk in the models (Fig. 2), especially for dialysis risk prediction. On the one hand, this may be related to a difference in risk (more myocardial infarction) with higher event rates: Tsai et al. [9] observed only 7.1% acute kidney injury in the validation cohort, compared to 15.3% in our cohort. On the other hand, the CIN outcome definition of Mehran et al. [6] used here is slightly different from the one by Tsai et al. [9], which may explain an offset in calibration.

Calibration as one of the performance measures of risk models is an important aspect of individualized risk prediction since it refers to the ability of a risk model to precisely forecast the true risk of a patient and therefore may also be seen as an indicator of whether the risk model fits the test data well. In our study, we observed a graphically more accurate risk model calibration for the Mehran risk model compared to the NCDR risk model, while statistical testing rendered both models inaccurate regarding calibration. Volatile calibration performance is often observed in external risk model validation studies: both NCDR models were externally validated in a Japanese patient cohort with over 11,000 patients [29], with similarly good discrimination performance, but also offsets in calibration [29]. Previous work from our group also observed corrupted calibration [10, 30]. The methodical work of Matheny et al. [31] shows that volatile calibration in risk models in interventional cardiology is related to small changes in event risk and data assessment, while retained discrimination shows the stability of risk factors over time. They conclude that continuous recalibration of risk models is mandatory to achieve perfect calibration. Additionally, slight differences in endpoint definitions between CIN according to Mehran (used here [6]), AKIN (used in the original NCDR population [9]) and the contrast-induced acute kidney injury definition (according to KDIGO [32, 33]) may influence comparability of risk models regarding calibration.

Preprocedural risk assessment, as well as procedural and postprocedural risk management are critical stepping stones to improve patient outcomes. A major advantage of the NCDR risk models over the Mehran risk model is that both NCDR risk models allow calculation of risk from preprocedural characteristics—while Mehran is a postprocedural risk prediction model. Knowing the risk of CIN or dialysis before the procedure, the operator may undertake preventive measures (—periprocedural risk management). The clinical indication of the procedure can be critically reviewed and high-risk patients may benefit from more intense or prolonged postprocedural monitoring of urine output and retention parameters. Contrast media volume is a key factor for the development of CIN, especially in high-risk patients [34] and may be managed accordingly: the use of biplane angiography systems helps to reduce contrast media exposure [35]. There are technical devices which are efficient in reducing contrast media over-injection [36]. The use of intravascular ultrasound (IVUS) and physiological guidance [37] may help in decreasing contrast media volume exposure for high-risk patients. Periprocedural risk management also includes volume expansion, which is still common practice (European class IIa recommendation for preprocedural hydration with isotonic saline [14]), although several studies showed no significant difference in the rate of CIN in patients with and without prophylactic hydration [38, 39]. Other studies investigated the influence on statin administration for the prevention of contrast-induced nephropathy with divergent results [40, 41]. Taken together, knowing patient risk for CIN upfront may hold the key for initiating adequate preventive measures in high-risk patients. Future studies are needed to investigate potential outcome benefits from preprocedural risk assessment and periprocedural risk management.

Major limitations of our study are the retrospective single-center design, which limits generalizability to other patient cohorts, and the limited number of included patients and events. Therefore, results, especially for the outcome ‘need for dialysis’, have to be interpreted with caution, as they are not clearly in favor of the NCDR model. A further limitation is missing data and the simple method of data imputation, especially for the Mehran model. However, sensitivity analysis in patients with complete data verified the overall results. There were almost no missing data for the calculation of the NCDR score, which underlines its usefulness as a preprocedural risk model.

Prospective validation of both risk models in a contemporary patient cohort is necessary to verify our results and help to further improve CIN risk management.

## Conclusion

In German patients undergoing coronary angiography, the modern NCDR risk model for predicting contrast-induced nephropathy showed superior discrimination performance compared to the established Mehran risk model. Both risk models showed inaccurate calibration, with slight advantages for the Mehran over the NCDR risk model for predicting contrast-induced nephropathy. Results for the outcome of ‘need for dialysis’ were equivocal. Further prospective studies are necessary to investigate patient outcome benefits from optimization in risk assessment and risk management.

## Supplementary Information

Below is the link to the electronic supplementary material.

Supplementary file1 (DOCX 33 KB)

## Abbreviations

AKI: Acute kidney injury
AUC: Area under curve
CI: Confidence-interval
CIN: Contrast-induced nephropathy
ROC: Receiver operating characteristic
PCI: Percutaneous coronary intervention
NSTEMI: Non-ST-segment elevation myocardial infarction
NRI: Net reclassification improvement
STEMI: ST-segment elevation myocardial infarction
