# Supplementary material for: National Cardiovascular Data Registry-Acute Kidney Injury (NCDR) vs. Mehran risk models for prediction of contrast-induced nephropathy and need for dialysis after coronary angiography in a German patient cohort
Source: J Nephrol. 2021 Aug 7;34(5):1491–500. doi: 10.1007/s40620-021-01124-9 (PMC8494719; doi:10.1007/s40620-021-01124-9)
Supplement: Supplementary file 1 — Supplementary file1 (DOCX 33 KB) [file 40620_2021_1124_MOESM1_ESM.docx]

**Supplementary material**

**Supplementary Table 1 – National Cardiovascular Data Registry risk model for prediction of acute kidney injury (NCDR-AKI)**

| **Age** | <50 | 50-59 | 60-69 | 70-79 | 80-89 | ≥90 |  | **NCDR-AKI** | |
| --- | --- | --- | --- | --- | --- | --- | --- | --- | --- |
|  | 0 | 2 | 4 | 6 | 8 | 10 |  | **Points** | **Risk %** |
| **Prior 2 weeks heart failure** | No | Yes |  |  |  |  |  | 0 | 1.9 |
|  | 0 | 11 |  |  |  |  |  | 5 | 2.6 |
| **Glomerular filtration rate (ml/min)** | >60 | 45-60 | 30-45 | <30 |  |  |  | 10 | 3.6 |
|  | 0 | 3 | 8 | 18 |  |  |  | 15 | 4.9 |
| **Diabetes** | No | Yes |  |  |  |  |  | 20 | 6.7 |
|  | 0 | 7 |  |  |  |  |  | 25 | 9.2 |
| **Prior heart failure** | No | Yes |  |  |  |  |  | 30 | 12.4 |
|  | 0 | 4 |  |  |  |  |  | 35 | 16.5 |
| **Prior cardiovascular disease** | No | Yes |  |  |  |  |  | 40 | 21.7 |
|  | 0 | 4 |  |  |  |  |  | 45 | 27.9 |
| **NSTEMI / STEMI** | No | NSTEMI / UA | STEMI |  |  |  |  | 50 | 35.1 |
|  | 0 | 6 | 15 |  |  |  |  | 55 | 43.0 |
| **Prior cardiogenic shock** | No | Yes |  |  |  |  |  | >60 | 51.4 |
|  | 0 | 16 |  |  |  |  |  |  |  |
| **Prior cardiac arrest** | No | Yes |  |  |  |  |  |  |  |
|  | 0 | 8 |  |  |  |  |  |  |  |
| **Anemia** | No | Yes |  |  |  |  |  |  |  |
|  | 0 | 10 |  |  |  |  |  |  |  |
| **Intra-aortic balloon pump** | No | Yes |  |  |  |  |  |  |  |
|  | 0 | 11 |  |  |  |  |  |  |  |

Suppl. Tab. 1: Risk model parameters and point assignment of the NCDR-AKI risk model for prediction of acute kidney injury [9]. NSTEMI = non-ST-segment elevation myocardial infarction; STEMI = ST-segment elevation myocardial infarction; UA = unstable angina pectoris.

**Supplementary Table 2 – National Cardiovascular Data Registry risk model for prediction of need for dialysis (NCDR-AKI-D)**

| **Prior 2 weeks heart failure** | No | Yes |  |  |  | **NCDR-AKI-D** | |
| --- | --- | --- | --- | --- | --- | --- | --- |
|  | 0 | 2 |  |  |  | **Points** | **Risk %** |
| **Glomerular filtration rate (ml/min)** | >60 | 45-60 | 30-45 | <30 |  | 0 | 0.03 |
|  | 0 | 1 | 3 | 5 |  | 1 | 0.05 |
| **Diabetes** | No | Yes |  |  |  | 2 | 0.09 |
|  | 0 | 1 |  |  |  | 3 | 0.15 |
| **NSTEMI / STEMI** | No | NSTEMI / UA | STEMI |  |  | 4 | 0.27 |
|  | 0 | 1 | 2 |  |  | 5 | 0.48 |
| **Prior cardiac arrest** | No | Yes |  |  |  | 6 | 0.84 |
|  | 0 | 3 |  |  |  | 7 | 1.5 |
|  |  |  |  |  |  | 8 | 2.6 |
|  |  |  |  |  |  | 9 | 4.4 |
|  |  |  |  |  |  | 10 | 7.6 |
|  |  |  |  |  |  | 11 | 12.6 |
|  |  |  |  |  |  | 12 | 20.3 |
|  |  |  |  |  |  | 13 | 31.0 |

Suppl. Tab. 2: Risk model parameters and point assignment of the NCDR-AKI-D risk model for prediction of need for dialysis. NSTEMI = non-ST-segment elevation myocardial infarction; STEMI = ST-segment elevation myocardial infarction; UA = unstable angina pectoris;

**Supplementary Table 3 – Mehran risk model**

| **Hypotension** | No | Yes |  |  |  | **Score** | **Risk of CIN %** | **Risk of Dialysis %** |
| --- | --- | --- | --- | --- | --- | --- | --- | --- |
|  | 0 | 5 |  |  |  | ≤5 | 7.5 | 0.04 |
| **Intra-aortic balloon pump** | No | Yes |  |  |  | 6-10 | 14.0 | 0.12 |
|  | 0 | 5 |  |  |  | 11-16 | 26.1 | 1.09 |
| **Congestive heart failure** | No | Yes |  |  |  | ≥16 | 57.3 | 12.6 |
|  | 0 | 5 |  |  |  |  |  |  |
| **Age > 75 years** | No | Yes |  |  |  |  |  |  |
|  | 0 | 4 |  |  |  |  |  |  |
| **Anemia** | No | Yes |  |  |  |  |  |  |
|  | 0 | 3 |  |  |  |  |  |  |
| **Diabetes** | No | Yes |  |  |  |  |  |  |
|  | 0 | 3 |  |  |  |  |  |  |
| **Contrast media volume** | Each 100 cc^3^ |  |  |  |  |  |  |  |
|  | 1 |  |  |  |  |  |  |  |
| **Serum creatinine < 1,5 mg/dl** | No | Yes |  |  |  |  |  |  |
|  | 0 | 4 |  |  |  |  |  |  |
| **OR** |  |  |  |  |  |  |  |  |
|  |  |  |  |  |  |  |  |  |
| **Estimated glomerular filtration rate (ml/min)** | >60 | 40-60 | 20-40 | <20 |  |  |  |  |
|  | 0 | 2 | 4 | 6 |  |  |  |  |

Suppl. Tab. 3: Risk model parameters and point assignment of the Mehran risk model for prediction of contrast induced nephropathy and need for dialysis. CIN = contrast-induced nephropathy.

**Supplementary Table 4 – Reclassification table for the comparison of NCDR-AKI vs. Mehran risk models for prediction of contrast-induced nephropathy**

| Total cohort (n = 2,067) | | | | | | | | | | | | | |
| --- | --- | --- | --- | --- | --- | --- | --- | --- | --- | --- | --- | --- | --- |
| No events (n = 1,750) | | | | | | |  | Events (n = 317) | | | | | |
|  |  | | | Reclassified | |  |  |  | | | Reclassified | |  |
|  | NCDR | | | Increased risk | Decreased risk | Net correctly reclassified (%) |  | NCDR | | | Increased risk | Decreased risk | Net correctly reclassified (%) |
| Mehran | 0-10% | 10-20% | 20-100% |  |  |  | Mehran | 0-10% | 10-20% | 20-100% |  |  |  |
| 0-10% | 748 | 27 | 6 |  |  |  | 0-10% | 43 | 5 | 5 |  |  |  |
| 10-20% | 371 | 99 | 41 | 74 | 646 | 32.7 | 10-20% | 56 | 26 | 21 | 31 | 115 | -26.5 |
| 20-100% | 147 | 128 | 183 |  |  |  | 20-100% | 15 | 44 | 102 |  |  |  |
| Net reclassification improvement (categorical) (95% CI) = 0.062 (-0.012 - 0.135); p = 0.10 | | | | | | | | | | | | | |
| Net reclassification improvement (continuous) (95% CI) = 0.221 (0.121 - 0.32); p < 0.01 | | | | | | | | | | | | | |

Suppl. Tab. 4: Reclassification table comparing NCDR and Mehran risk models for prediction of contrast-induced nephropathy in all patients. P < 0.05 was considered statistically significant.

**Supplementary Table 5 – Reclassification table for the comparison of NCDR-AKI-D vs. Mehran risk models for prediction of need for dialysis**

| Total cohort (n = 2,067) | | | | | | | | | | | | | |
| --- | --- | --- | --- | --- | --- | --- | --- | --- | --- | --- | --- | --- | --- |
| No events (n = 2,036) | | | | | | |  | Events (n = 31) | | | | | |
|  |  | | | Reclassified | |  |  |  | | | Reclassified | |  |
|  | NCDR | | | Increased risk | Decreased risk | Net correctly reclassified (%) |  | NCDR | | | Increased risk | Decreased risk | Net correctly reclassified (%) |
| Mehran | 0-1% | 1-5% | 5-100% |  |  |  | Mehran | 0-1% | 1-5% | 5-100% |  |  |  |
| 0-1% | 1418 | 17 | 1 |  |  |  |  | 10 | 1 | 0 |  |  |  |
| 1-5% | 383 | 38 | 0 | 18 | 559 | 26.6 |  | 5 | 2 | 0 | 1 | 18 | -54.8 |
| 5-100% | 98 | 78 | 2 |  |  |  |  | 3 | 10 | 0 |  |  |  |
| Net reclassification improvement (categorical) (95% CI) = -0.283 (-0.48 - -0.085); p < 0.01 | | | | | | | | | | | | | |
| Net reclassification improvement (continuous) (95% CI) = -0.163 (-0.502 - 0.177); p = 0.35 | | | | | | | | | | | | | |

Suppl. Tab. 5: Reclassification table comparing NCDR and Mehran risk models for prediction of need for dialysis in all patients. P < 0.05 was considered statistically significant.
